# Facile Synthesis of Thermoplastic Polyamide Elastomers Based on Amorphous Polyetheramine with Damping Performance

**DOI:** 10.3390/polym13162645

**Published:** 2021-08-09

**Authors:** Jie Jiang, Qiuyu Tang, Xun Pan, Jinjin Li, Ling Zhao, Zhenhao Xi, Weikang Yuan

**Affiliations:** 1 State Key Laboratory of Chemical Engineering, School of Chemical Engineering, East China University of Science and Technology, Shanghai 200237, China; jjiangecust@gmail.com (J.J.); qiuyu.tang@foxmail.com (Q.T.); lijinjin@ecust.edu.cn (J.L.); zhaoling@ecust.edu.cn (L.Z.); wkyuan@ecust.edu.cn (W.Y.); 2 Flinders Institute for Nanoscale Science and Technology, Flinders University, Sturt Road, Bedford Park, SA 5042, Australia; caroline.pan@flinders.edu.au; 3 College of Chemistry and Chemical Engineering, Xinjiang University, Urumqi 830046, China

**Keywords:** one-pot method, polyamide, elastomer, PPG diamine, damping property

## Abstract

Novel thermoplastic polyamide elastomers (TPAEs) consisting of long-chain semicrystalline polyamide 1212 (PA1212) and amorphous polyetheramine were synthesized via one-pot melt polycondensation. The method provides accessible routes to prepare TPAEs with a high tolerance of compatibility between polyamide and polyether oligomers compared with the traditional two-step method. These TPAEs with 10 wt % to 76 wt % of soft content were obtained by reaction of dodecanedioic acid, 1,12-dodecanediamine, and poly(propylene glycol) (PPG) diamine. The structure–property relationships of TPAEs were systematically studied. The chemical structure and the morphologic analyses have revealed that microphase separation occurs in the amorphous region. The TPAEs that have long-chain PPG segments consist of a crystalline polyamide domain, amorphous polyamide-rich domain, and amorphous polyetheramine-rich domain, while the ones containing short-chain PPG segments comprise of a crystalline polyamide domain and miscible amorphous polyamide phase and amorphous polyetheramine phase due to the compatibility between short-chain polyetheramine and amorphous polyamide. These novel TPAEs show good damping performance at low temperature, especially the TPAEs that incorporated 76 wt % and 62 wt % of PPG diamine. The TPAEs exhibit high elastic properties and low residual strain at room temperature. They are lightweight with density between 1.01 and 1.03 g/cm^3^. The long-chain TPAEs have well-balanced properties of low density, high elastic return, and high shock-absorbing ability. This work provides a route to expand TPAEs to damping materials with special application for sports equipment used in extremely cold conditions such as ski boots.

## 1. Introduction

Thermoplastic elastomers (TPEs) are usually multiblock segmented copolymers consisting of thermodynamic incompatible hard blocks and soft blocks, normally leading to a microphase separated structure. The soft blocks are often polyether or aliphatic polyester with low glass transition temperature (*T*_g_), enabling elastomeric properties to the copolymers. Moreover, the hard blocks containing crystalline units form a physical cross-linked site. Chemical covalent links between the two blocks can avoid macro-phase separation. These materials bridge the gap between thermoplastics and elastomers. Thermoplastic polyurethanes (TPUs), thermoplastic polyether ester elastomers (TPEEs), and thermoplastic polyamide elastomers (TPAEs) are the three main types of segmented TPEs. TPEs have received much industrial and commercial interest duo to their good thermomechanical properties, excellent chemical resistance, wide service temperature range, and ease of processing.

TPAEs have been recently developed as a relatively new family of engineering plastics because of their highly efficient energy return, good performance at low temperature, and selective gas permeability. They are widely used in sports equipment, such as ski boots, cleated shoes, and running shoes. Generally, TPAEs are segmented poly(ether-*block*-amide) copolymers composed of polyether as soft segments and polyamide as hard segments. The performance of the material depends not only on the ratio of soft and hard segments but also the nature of a soft block when using a certain polyamide. Currently, the main soft blocks for TPAEs are PEG [1] and PTMG, enabling antistatic property and good flexibility, respectively. Commercially available TPAEs have been mainly developed by Arkema and Evonik under the trade name of PEBAX and VESTAMID, respectively. The copolymers are synthesized by the polycondensation of carboxyl terminated polyamide (PA6, PA11, or PA12 oligomers) and hydroxyl terminated polyether (PTMG or PEG), resulting in ester linkage [2,3], which is the so-called “two-step method”. The structure–property relationships of TPAEs have been extensively studied [4,5,6,7,8,9,10]. For example, TPAEs consisting of PTMG has been mostly used for footwear [11,12,13,14] and gas separation [15,16], while those containing PEG as a flexible phase are employed to selectively separate CO_2_ [17,18,19,20] and permanent antistatic packaging [21]. It should be noted that PTMG and PEG are a semicrystalline C4 building block and C2 building block, respectively. The C3 building block has been hardly used in the preparation of TPAEs. In our previous work, a linear semicrystalline poly(trimethylene glycol) (PPDO) has been utilized to prepare TPAEs with high elasticity [22]. Nevertheless, for these semicrystalline blocks, the melting of the corresponding segments will cause the variation of storage modulus, which is not conducive to the use of materials in this temperature range [2]. To enrich the TPAE family, poly(propylene glycol) (PPG, C3) is an alternative amorphous polyether with a methyl side group on each repeat unit, which has weak dispersion and a low dipole moment, resulting in the superior elasticity to polyurethane elastomers [23,24]. However, the reactivity of secondary hydroxyl groups in PPG is relatively low, and its poor compatibility with polyamide oligomers prevents the formation of high molecular weight copolymers. Considering the condensation of hydroxyl or amino groups with carboxyl groups, PPG diamine (trade name Jeffamine D) is an alternative C3 building block. It is a non-crystalline aliphatic polyetheramine with low viscosity and low tendency to form a hydrogen bond, which is generally used as a gasoline additive, curing agents of epoxy resins [25,26], unit of epoxy-imine vitrimer [27], precursor of main-chain polybenzoxazines resin [28], or soft segments of Pus [29], providing high toughness, thermal stability, or flexibility. For PPG-based TPAEs, monomer casting PA6 modified with maximum 6 wt % of this polyetheramine shows an obvious decrease in crystallization ability and an elastic deformation behavior [30]. Grymans et al. selected PA46 salt and PPG diamine to prepare PA46-PPG segmented copolymers via condensation followed by solid-phase polycondensation [31]. However, severe macro-phase separation occurs despite adding *m*-cresol as solvent. The solid products are inhomogeneous after post-polymerization when using mole mass 2000 of Jeffamine D. Jo et al. synthesized PA6-PPG copolymers with soft content from 10 wt % to 56 wt % by melt copolymerization [32,33,34], whose microphase separation structure was systematically investigated. Shibasaki et al. reported a monodisperse poly(N-methyl benzamide) copolymerized with PPG diamine in solution to afford non-hydrogen-bondable block copolymers, which are non-crystalline materials but showing weak tensile modulus [35]. Luo et al. prepared fluorene-based PA-PPG membranes via solution polycondensation with high carbon dioxide permeability [36,37].

It is noteworthy that for the sporting applications of TPAEs, the attenuation of vibration is an important property. The methyl side group in the PPG backbone allows strong energy dissipation by internal friction, enabling the shock-absorbing capacity of the TPAEs when using them as potential damping materials, which has not been extensively investigated. PA1212 is an important semicrystalline engineering plastic, which owns high toughness and strength, low temperature resistance, and low shrinkage [38]. It has close properties with PA12, and the polymerization of PA1212 does not require high temperature and high pressure of PA12, which is mostly used to produce commercial PEBAX. Furthermore, it is not easy to prepare the high molecular weight of TPAEs using the “two-step method” due to the incompatibility between polyamide and polyether oligomer and the difficulties in adjusting the stoichiometric balance of reactive groups [39]. In this work, one-pot melt polycondensation was proposed to firstly synthesize a series of segmented TPAEs with variable compositions of PA1212 block (C12) and PPG block (C3). The TPAEs were prepared directly from monomers of PA1212 and PPG diamine. This procedure is feasible with the advantages of low starting viscosity, ease of stoichiometric balance of acid and amine groups, and high tolerance of compatibility between polyamide and polyether compared with the two-step method. This one-pot strategy is its simplicity, high efficiency, and low cost, especially using commercial PPG diamine (C3) to manufacture advanced materials. The chemical structures of novel TPAEs were studied using nuclear magnetic resonance (NMR) and Fourier transform infrared spectroscopy (FT-IR). The microphase-separated structures were characterized by differential scanning calorimetry (DSC), dynamic thermomechanical analysis (DMA), and X-ray diffraction and confirmed by microscopy technics. Additionally, tensile tests and cyclic tensile tests were performed to evaluate the mechanical properties of the series of new copolymers, indicating that the synthesized TPAEs have high elasticity. The novel TPAEs have the damping properties at low temperature, especially those with high flexible content due to the incorporation of the amorphous PPG segment. The effects of content and length of the PPG segment on microphase separated morphology were systematically investigated. Thermal gravimetric analysis (TGA) was applied to find that the TPAEs have good thermal stability. These novel family of TPAEs are believed to have great potential for low-temperature applications.

## 2. Experiment Section

### 2.1. Materials

Dodecanedioic acid (≥99%), deuterated trifluoroacetic acid (TFA-d, 99.5 atom %D), *m*-cresol (≥99%), and 1,1,1,3,3,3-Hexafluoro-2-propanol (HFIP, 99%) were purchased from Shanghai Titan Science and Technical Company (Shanghai, China). Polyetheramine JeffamineD400 (Mn ≈ 400 g·mol^−1^) and JeffamineD2000 (Mn ≈ 2000 g·mol^−1^) (PPG diamine) were purchased from Shanghai Aladding Biochemical Technology Co., Ltd. (Shanghai, China), and 1,12-dodecanediamine (≥99%) was supplied by Zibo Guangtong Chemical Company (Shandong, China). Sodium hypophosphite monohydrate (NaH_2_PO_2_·H_2_O, ≥98%) was purchased from Sinopharm Chemical Reagent Co., Ltd. (Shanghai, China). All the reagents were used as received without further purification.

### 2.2. Characterization

#### 2.2.1. Intrinsic Viscosity

The intrinsic viscosity [*η*] of TPAEs was determined by an Ubbelohde viscometer using *m*-cresol as solvent with a concentration of 0.5 g·dL^−1^ at 25 ± 0.01 °C. [*η*] was calculated by the following Solomon—Ciuta equation of a single-point method (Equation (1)).

(1) $$\left[\eta \right]=\sqrt{2\left(\eta_{sp}-ln\eta_{r}\right)}/c$$

where *η_sp_* is specific viscosity, *η_r_* is relative viscosity, and *c* is the TPAE concentration (g·dL^−1^).

#### 2.2.2. Density Measurement

The density of samples was determined using a water displacement method according to *ASTM D792-13*; the density (*ρ*) was calculated by Equation (2):

(2) $$\left[\rho \right]=\left(\frac{a}{a-b}\right)\rho_{water}$$

where *a* is the apparent mass of sample in air, *b* is the apparent mass of sample completely immersed in water, and *ρ_water_* is the density of water.

#### 2.2.3. Nuclear Magnetic Resonance (NMR)

The TPAEs samples (15 mg) were dissolved in 0.5 mL of deuterated trifluoroacetic acid (TFA-d). ^1^H NMR and 2D NMR spectra were recorded on an AVANCE-600 spectrometer (Bruker Ascend, 600 MHz, Germany) at ambient temperature using TFA-d as solvent. The chemical shift of the solvent is 11.6 ppm. Two-dimensional (2D) correlation spectroscopy (^1^H-^1^H COSY) using the standard Bruker pulse program cosygpppqf with the following parameters: spectral width SW1 = SW2 = 11,904.7 Hz, acquisition time 0.086 s, relaxation delay 2.0 s; processing, SI = 1024 (f2, f1), WDW = QSINE.

#### 2.2.4. Fourier Transform Infrared Spectroscopy (FT-IR)

The films of TPAE were analyzed on a Nicolet 6700 spectrometer (Thermo Fisher Scientific, USA) equipped with ZnSe crystal using an attenuated total reflectance (ATR) model. Films were prepared by drop-casting from HFIP and kept at room temperature for 48 h followed by drying in vacuum at 60 °C for another 48 h to remove the solvent. The spectra were recorded from 4000 to 650 cm^−1^ with a spectral resolution of 4 cm^−1^.

#### 2.2.5. Gel Permeation Chromatography (GPC)

GPC was performed using HFIP as eluent at 40 °C on an Agilent PL-GPC50 equipped with a differential refractive index detector. Two PLHFIPgel columns (300 × 7.5 mm, 9 μm) were used. The sample concentration and eluent flow rate were 3 mg·mL^−1^ and 1.0 mL·min^−1^, respectively. Calibration of the measurements was carried out with poly(methyl methacrylate) standards.

#### 2.2.6. Differential Scanning Calorimetry (DSC)

Thermal properties of TPAEs were characterized on a Q2000 DSC apparatus (TA instrument, USA). The instrument was calibrated with indium before measurements. A sample was heated to 200 °C at a rate of 10 °C·min^−1^ and held for 3 min under nitrogen atmosphere to eliminate any thermal history. Then, the sample was cooled to −80 °C and reheated to 200 °C at a cooling/heating rate of 10 °C·min^−1^. The second endothermal curve was recorded to analyze the thermal behavior. The *Χ*_c_ crystalline fraction referring to the amount of PA1212 was calculated according to Equation (3):

(3) $$X_{c}=∆H_{m}/(w_{PA}∆H_{m}^{0})$$

where the ideal enthalpy of fusion of pure PA1212 $∆H_{m}^{0}$ = 292.2 J/g [40].

#### 2.2.7. Wide-Angle X-ray Diffraction (WAXD)

The WAXD measurements were implemented on an X-ray diffractometer (Bruker D8 Advance, Germany) equipped with a Cu K_α_ radiation source (wavelength *λ* = 0.1542 nm) at ambient temperature. The diffraction patterns were recorded in the range of 2θ from 5° to 80°. Samples with about 0.5 mm of thickness were prepared by casting from HFIP.

#### 2.2.8. Small-Angle X-ray Scattering (SAXS)

Films of TPAEs casting from HFIP solution with about 0.5 mm in thickness were used for the tests. Samples were subsequently annealed under vacuum at 80 °C for 12 h to avoid the influence of process history on the morphology. SAXS profiles were obtained from a SAXSess mc2 apparatus (Anton Paar, Austria) equipped with a Cu K_α_ monochromatic radiation (wavelength *λ* = 0.15406 nm). The measurements were operated at 40 kV and 30 mA at ambient temperature. The scattering intensity (*I*) was obtained as a function of scattering vector (*q*).

#### 2.2.9. Atomic Force Microscopy (AFM)

AFM (Bruker ICON, Germany) was used to investigate the morphology of TPAEs in tapping mode with an Al reflective coated silicon probe (RTESPA, cantilever: T × L × W = 3.4 × 125 × 40 μm^3^, 300 kHz, 40 N·m^−1^). NanoScope Analysis software was employed for the data processing. Samples were dissolved dimethylacetamide (DMAc) to acquire 0.2 wt % of polymer solution. Thin films were obtained by drop casting on the silicon wafer followed by carefully evaporating the solvent above 10 °C of the crystalline temperature of the samples. Then, all the wafers were immediately isothermal treated for 20 min.

#### 2.2.10. Transmission Electron Microscopy (TEM)

The morphology observation of TPAEs samples was performed on a transmission electron microscope (JEM-1400, Japan); 0.5 wt % sample solutions were prepared by dissolving the TPAEs in DMAc. Thin films were fabricated by dropping the solution on the copper mesh followed by carefully evaporating the solvent above 10 °C of the crystalline temperature of the samples. Then, all the samples were immediately isothermal treated for 20 min.

#### 2.2.11. Dynamic Thermomechanical Analysis (DMA)

Dynamic thermomechanical analysis was conducted on a DMA Q800 (TA Instruments, USA) equipped with a liquid nitrogen cooling apparatus. The specimens with a rectangular geometry (30 × 5 × 0.5 mm^3^) were prepared by casting from HFIP solution. The measurements were performed in the tension mode at a heating rate of 5 °C·min^−1^ from −100 to 100 °C in nitrogen atmosphere at a frequency of 1 Hz. The storage modulus (*E*’) and loss factor (*tan* *δ*) were recorded as a function of temperature for each sample. Furthermore, the temperature of the *tan δ* peak is used to define the glass transition temperature (*T*_g_) of each sample.

#### 2.2.12. Mechanical Properties

Mechanical testing was performed using dumbbell-shaped samples (35 × 2 × 0.5 mm^3^), according to the guideline of ISO 527-2), which were prepared by casting from HFIP and cut by a mold. The tensile testing was conducted on an Instron 3367 (USA) universal testing machine at a speed of 50 mm·min^−1^ at room temperature. Cyclic tensile tests were conducted on a CMT6103 testing machine (MTS, USA). The test specimens were repeatedly exposed to consecutive cycles to a constant strain of 100% (or 200%) with a constant speed of 50 mm·min^−1^. The recovery was measured by observing the residual strain after 10 cycles.

### 2.3. Synthesis of TPAEs

A typical one-pot synthesis procedure is described as follows. To a 250 mL three-necked flask equipped with a mechanical stirrer, an argon inlet, and a condenser, dodecanedioic acid (17.27 g, 0.075 mol), 1,12-dodecanediamine (7.52 g, 0.0375 mol), Jeffamine D2000 (75 g, 0.0375 mol), and sodium hypophosphite monohydrate (1 wt %) were added. The mixture was slowly heated to 220 °C and kept for 2.5 h in argon flow at a stirring speed of 200 rpm until no water condensation was observed. Afterwards, the vacuum was gradually applied to 100 Pa within 30 min. After 2 h of reaction, the reaction temperature was raised to 240 °C and the vacuum was kept constant at 80–100 Pa for another hour. The resulting product was poured into ice water and dried under reduced pressure.

## 3. Results and Discussion

### 3.1. Synthesis and Structure Characterization of TPAEs

**Scheme 1:**
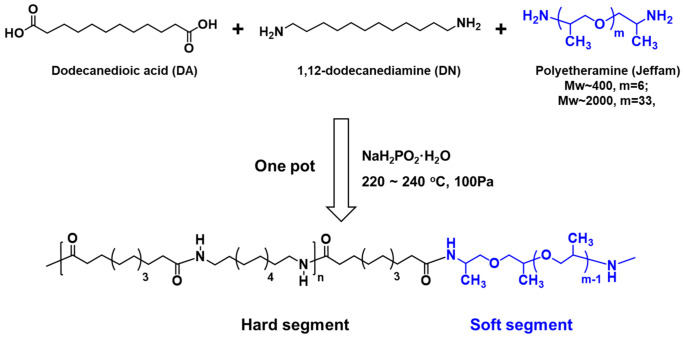
One-pot synthesis process of TPAEs based on PA1212 and polyetheramine.

The one-pot polycondensation for novel TPAEs was performed using monomers of PA1212 and polyetheramine in the presence of sodium hypophosphite monohydrate, as shown in Scheme 1. The molar amount of DA is equal to the sum of DN and PPG diamine to maintain the stoichiometric balance of functional groups. Dodecanedioic acid (DA) can react with both 1,12-dodecanediamine (DN) and PPG diamine, and the reaction of DA and DN produces the PA1212 segment. Practically, the equimolar of DA and DN forms PA1212 and the excess DA limits the molar weight of the PA1212 oligomer on one hand and links the PPG diamine segment on the other hand. These TPAEs can also be considered as copolyamides due to the amide linkage between polyamide and polyether, thus conferring more resistance to hydrolysis than the most reported TPAEs containing ester linkages. Six TPAEs with varied soft contents were synthesized by adjusting the feed ratio of dodecanedioic acid, 1,12-dodecanediamine, and polyetheramine. The polyetheramine used are long-chain Jeffamine D2000 and short-chain Jeffamine D400 with mole masses of approximately 2000 g·mol^−1^ and 400 g·mol^−1^, respectively, and the polyamide has a regular-length segment. The sample code was expressed as TPAE-x, where x corresponds to the soft content determined according to the ^1^H NMR spectra.

**Figure 1:**
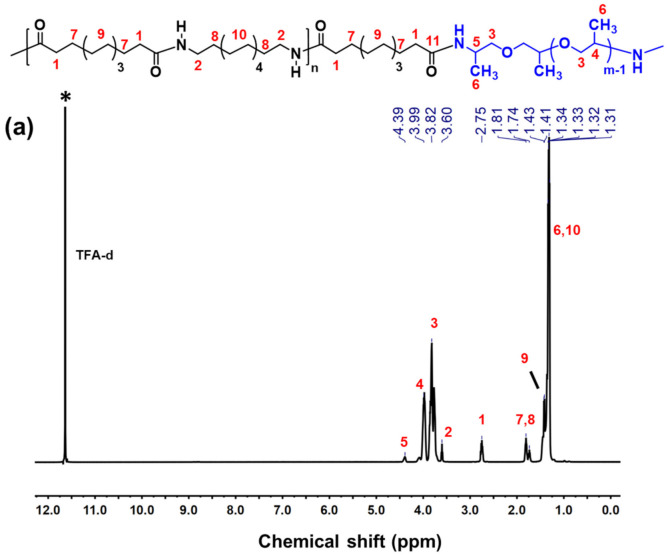

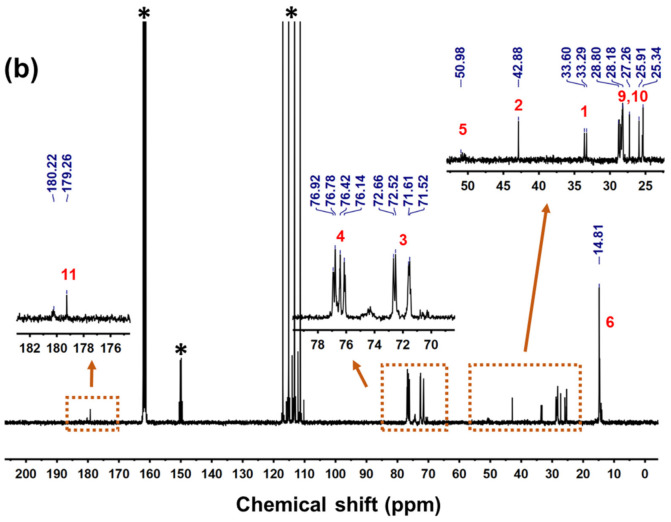
^1^H NMR (**a**) and ^13^C NMR (**b**) spectrum of TPAE-0.76 (* Solvent: TFA-d).

**Figure 2:**
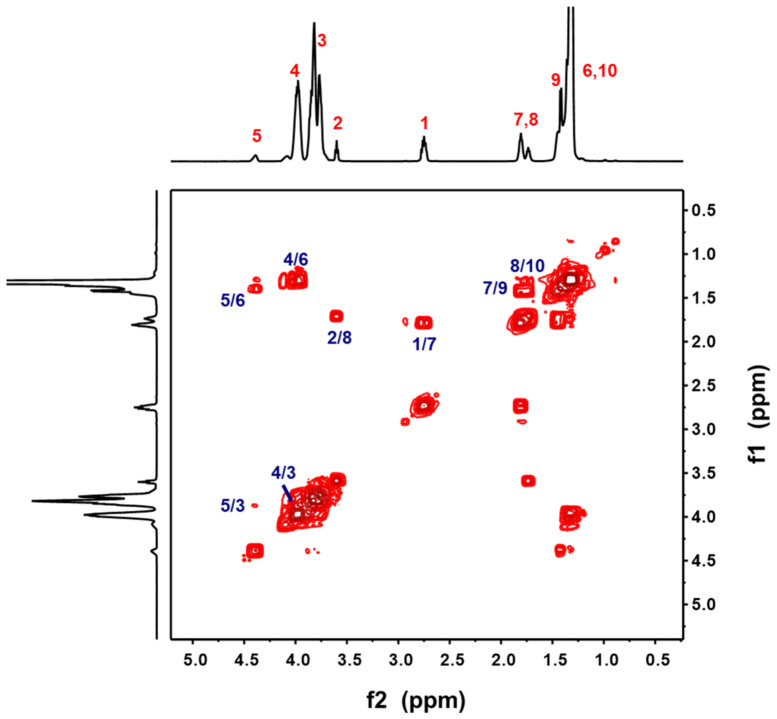
^1^H-^1^H COSY spectrum of TPAE-0.76.

The chemical structures of the TPAEs are confirmed by NMR and FT-IR. Figure 1a,b and Figure 2 depict the ^1^H NMR, ^13^C NMR, and ^1^H-^1^H COSY spectra of TPAE-0.76, respectively. Each peak is assigned to the corresponding hydrogen and carbon in TPAE. The characteristic peaks at δ = 2.75 ppm (H^1^) and δ = 3.60 ppm (H^2^) are attributed to CH_2_ proton next to the carboxyl and nitrogen atom respectively, indicating that the PA1212 segment has formed. The correspondingly CH_2_ carbons are found at δ = 33 ppm (C^1^) and δ = 42 ppm in the ^13^C spectrum, respectively. It is noteworthy that the appearance of resonance at δ = 4.39 ppm (H^5^) confirms that polyetheramine has been bonded to the PA1212 segment, which agrees with the results of PA6-based copolyetheramide [32]. The corresponding CH carbon appears at δ = 50 ppm. The peaks of CH_2_ proton (C^7^, C^8^, C^9^) in the aliphatic chain of PA1212 emerge at 1.31–1.81 ppm. The strong signal at 1.31 ppm is attributed to side CH_3_ (C^6^) of polyetheramine. The peaks at 3.82 ppm and 3.99 ppm belong to CH_2_ and CH of the polyetheramine backbone, respectively. The corresponding carbon resonance can be found in a ^13^C NMR spectrum. Thus, the soft content can be calculated according to peak areas of H^5^, H^2^, and H^3^ in ^1^H NMR [5,6], and the mass ratios of soft segment are listed in Table 1. The exact compositions of copolymers are close to theoretical calculation based on the feeding ratio, indicating the synthesized copolymers with expected structure. The slight decreases of soft contents calculated by NMR are because the hydrophilic polyetheramine was brought out with water vapor. It is noted that the peak at 180 ppm suggests that there are more residual carboxyl groups in TPAE than amine groups. ^1^H-^1^H COSY can demonstrate coupling relations of protons on adjacent carbon atoms, presenting cross dots on the spectrum. The cross-signals found in the COSY spectrum prove the presence of the protons in the copolymer backbone. The polyetheramine segment has been successfully bonded to the PA1212 segment confirmed by the cross-peaks at 5/6 and 5/3.

**Figure 3:**
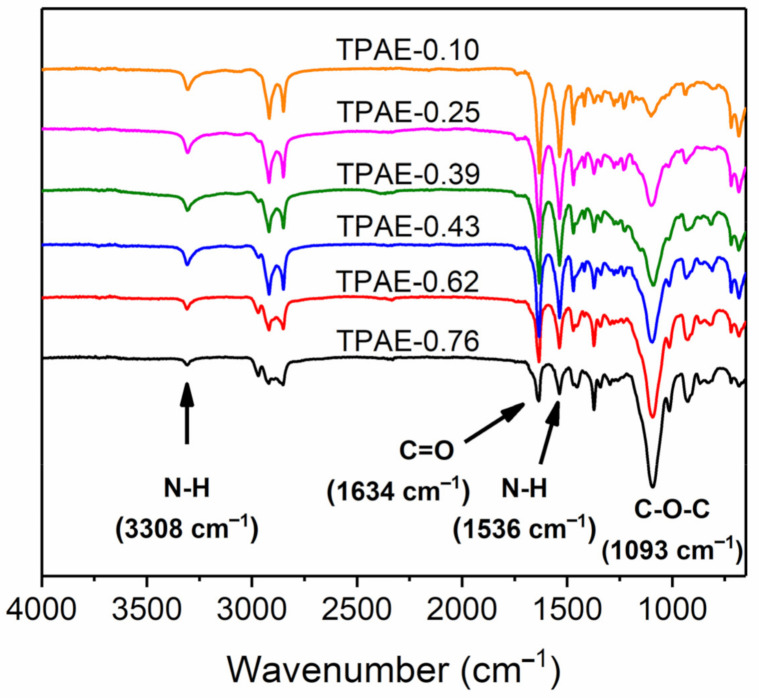
FT-IR spectra of TPAEs.

The structure analysis of TPAEs by FT-IR is presented in Figure 3. The characteristic peaks at 3308 cm^−1^ (amide A), 1634 cm^−1^ (amide I), and 1536 cm^−1^ (amide II) are attributed to the PA1212 segment [41]. The incorporation of the polyetheramine segment to the PA1212 backbone is confirmed by the distinct absorption band at 1093 cm^−1^ (C-O-C stretching vibration) that became more pronounced with increased soft content, which is ascribed to the polyetheramine segment [41]. The peaks at 2850–2970 cm^−1^ are assigned to the stretching vibration of -C-H-.

### 3.2. Thermal Characterization

**Figure 4:**
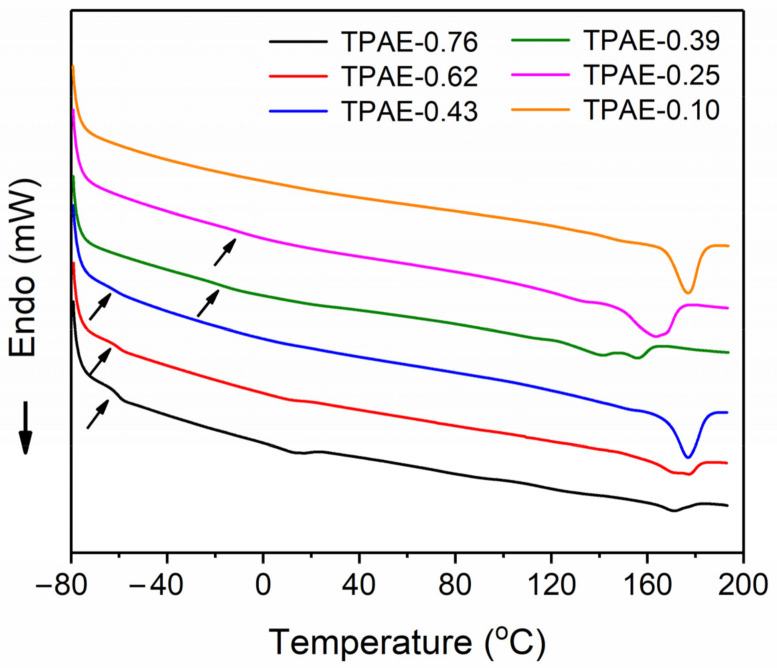
DSC thermograms of different TPAEs.

To gain a deep insight into the phase separation behavior and the crystal structure of the copolymers with the different block length of hard and soft segments, the endothermic behavior of different TPAEs was recorded by DSC testing and illustrated in Figure 4. The corresponding characteristic parameters are listed in Table 2.

First, the polyetheramine is completely amorphous, as confirmed by the DSC scan (Figure S1, Supplementary Material). It can be observed that each copolymer sample has only one endothermal peak during the heating scan, which is ascribed to the melting behavior of the semicrystalline polyamide segment. This is much different from the double crystalline TPAEs containing PTMG, PEG, or PPDO soft segments with two distinct endothermal peaks. The amorphous nature of PPG may endow high flexibility and elasticity. Furthermore, the copolymers having long-chain Jeffamine D2000 exhibit similar *T_g_* in spite of different compositions (TPAE-0.76, TPAE-0.62, and TPAE-0.43), which suggests that microphase separation occurs in the amorphous region. The *T_g_* values of the three samples are close to −69.5 °C of the pristine soft block (Figure S1, Supplementary Material). Differently, the copolymers containing short-chain Jeffamine D400 show increasing *T_g_* values with increasing polyamide content, which indicates that the flexible polyetheramine segments are compatible with the rigid polyamide segments in the amorphous region [33] (TPAE-0.39 and TPAE-0.25). However, in sample TPAE-0.10, the *T_g_* is not detected, suggesting the high miscibility between polyetheramine and polyamide.

For containing different chain lengths and content of polyetheramine, the melting temperature of TPAEs increases obviously as the hard content increases. It can be found that the segmented polyamide has a lower melting temperature than homo PA1212 (184.2 °C). The melting temperature of copolymers containing short-chain Jeffamine D400 show a relatively large difference to homo PA1212. It is noted that TPAE-0.43 and TPAE-0.39 have the similar hard content but distinct melting points. This is because short-chain Jeffamine D400 is miscible with polyamide and restricts the growth of polyamide crystals, resulting in a reduction of crystalline degree (from 22.6% to 19.6%). Therefore, it is an effective strategy to decrease the processing temperature of the elastomer by using short chains of polyether. Correspondingly, the crystallization temperature exhibits the same trend as melting temperature (Figure S2, Supplementary Material), which also indicates that soft blocks are compatible to the hard blocks in the amorphous region and that short chain polyetheramine is miscible in this amorphous region. Otherwise, the multiple melting endotherms are caused by (1) the melting of crystallites with different lamellae thickness, (2) the melting of different crystal forms, or (3) the crystallization and remelting of imperfect crystallites [33].

### 3.3. Morphological Characterizations

**Figure 5:**
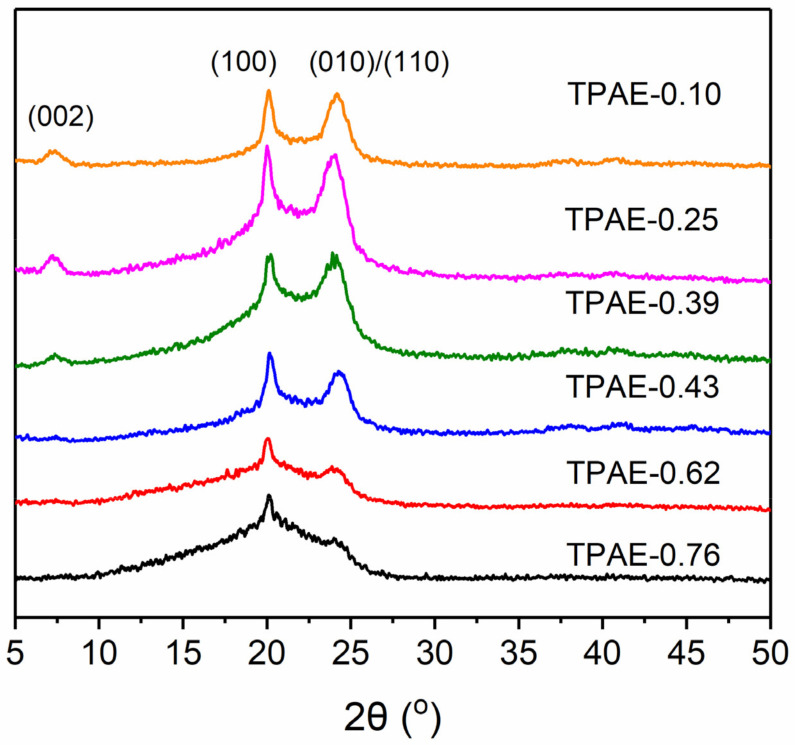
WAXD patterns of the TPAEs at ambient temperature.

The wide-angle X-ray diffraction of six TPAEs with different compositions are shown in Figure 5. The copolymer films with non-crystalline soft segments show two main diffraction peaks at around *2θ* = 20.1° and 24.0° that are attributed to the (100) crystal plane and (010)/(110) crystal plane [40,41], suggesting that the films adopt a triclinic α-crystal phase. The corresponding *d* spacing values are 0.44 nm and 0.37 nm, which represent the inter-chain distance within the hydrogen bonded-sheet and the inter-sheet distance, respectively [42]. The microphase separation takes place in the amorphous region of novel TPAEs.

**Figure 6:**
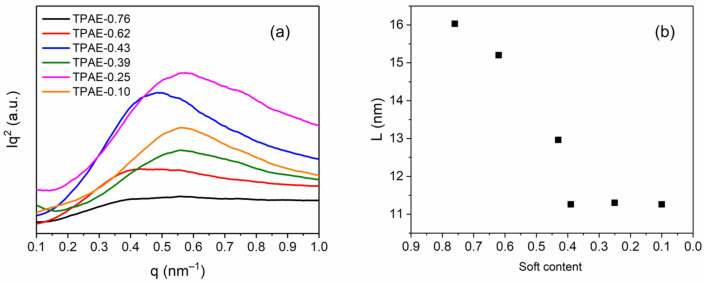
(**a**) Lorentz corrected SAXS profiles of the TPAEs and (**b**) long period vs. soft contents.

Small-angle X-ray scattering was conducted to further explore the microphase separated structure of the TPAEs. Figure 6a presents the Lorentz corrected SAXS profiles (*Iq*^2^ vs. *q*) of the TPAEs with different compositions. According to Bragg’s law, the corresponding long period (*L*) can be calculated based on the maximum value of the scattering vector (*q_max_*), *L* = 2π/*q_max_,* and the results are illustrated in Figure 6b. Only one broad scattering peak appears in all the TPAEs samples in Figure 6a. The peak indicates the existence of a periodic structure. The intensity peaks of copolymers with Jeffamine D400 (TPAE-0.39, TPAE-0.25, and TPAE-0.10) are similarly broad, while the ones with Jeffamine D2000 (TPAE-0.76, TPAE-0.62, and TPAE-0.43) are broadened as the soft content increases, suggesting the existence of a long periodic structure in copolymers with Jeffamine D2000, and the structure disappears as soft content increases. Furthermore, due to the non-crystallization of soft segments, the scattering peaks are attributed to the repeat distance between the polyamide crystalline domains. The *L* values decrease from 16.03 nm of TPAE-0.76 to 11.26 nm of TPAE-0.10 with increasing soft content and stay close when copolymers have short-chain Jeffamine D400. The reduction of *L* in value is owing to the crystallization of polyamide [43,44]. It is proof that short-chain polyetheramine is miscible with polyamide amorphous domain.

**Figure 7:**
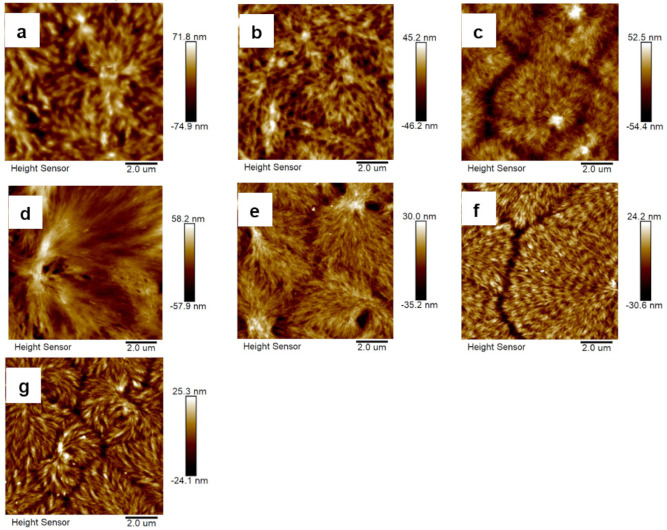
AFM height images of drop-casting TPAEs: (**a**) TPAE-0.76, (**b**) TPAE-0.62, (**c**) TPAE-0.43, (**d**) TPAE-0.39, (**e**) TPAE-0.25, (**f**) TPAE-0.10, and (**g**) homo PA1212.

The surface topographical characteristic of the TPAEs with different compositions was investigated by AFM at ambient temperature, and the height images are shown in Figure 7. The AFM height image of homo PA1212 is included for comparison. The copolymers display well-ordered crystalline structure and the spherulites of the polyamide domain with a clear boundary can be found except for the high soft content of TPAE-0.76 and TPAE-0.62. It should be noteworthy; however, these two copolymers contain a polyamide crystal phase according to DSC and WAXD results. The slender and rigid polyamide domain is observed, which can be explain by the assumption that the polyamide crystalline domain forms randomly ordered lamellar crystals by the dilute solution casting method [5]. For the other samples, the spherulitic morphology consisting of crystalline polyamide and amorphous polyamide segments are well dispersed in the amorphous polyetheramine. It is believed that the amorphous polyetheramine chains are comprised of the spherulites or filled among the lamellar crystals [41].

In addition, the non-crystalline soft segments have a distinguishing effect on the morphology features. It can be found that when using long-chain Jeffamine D2000, the spherulitic structure forms only in TPAE-0.43 with relative high polyamide content, showing packed down-feather-like lamellar topography; for TPAE-0.39 to TPAE-0.10 with short-chain Jeffamine D400, the bundle-like structure with filamentous polyamide crystals turns into similar spherulite with a feather-like structure. Furthermore, with the increasing of polyamide content, the short-chain polyetheramine has a much greater dilution effect on the crystallization of polyamide than the long-chain polyetheramine because of the difference in the compatibility of distinct polyetheramines with polyamide. As a result, the degree of microphase separation of copolymers with long-chain soft segments is higher than that with short chain. The above deductions are further validated from the polarized optical microscopy (POM) images (Figure S3, Supplementary Material). Only very small crystals can be found in TPAEs with low amount of soft content (containing short-chain polyetheramine), and small size and sparse packed spherulites of polyamidecan be observed in TPAE-0.10.

**Figure 8:**
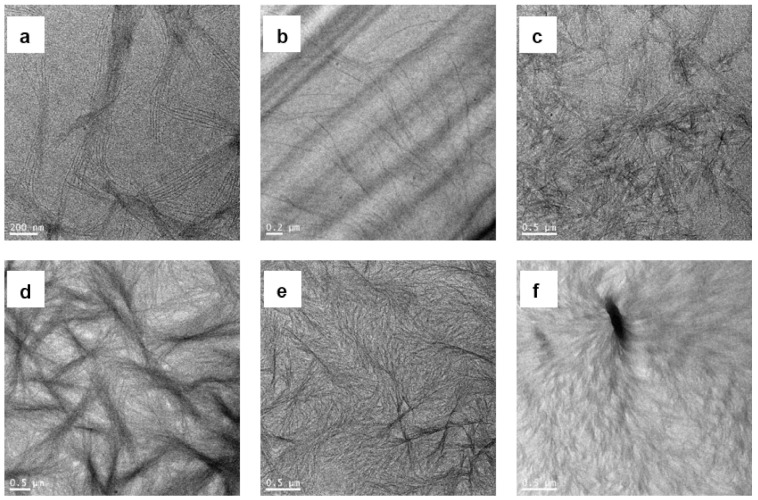
TEM images of (**a**) TPAE-0.76, (**b**) TPAE-0.62, (**c**) TPAE-0.43, (**d**) TPAE-0.39, (**e**) TPAE-0.25, and (**f**) TPAE-0.10.

To further investigate the morphological evolution with different compositions of the TPAEs, TEM was performed, and the representative images are shown in Figure 8. The TPAE-0.76 and TPAE-0.62 with high soft content grow long rigid-rod polyamide crystals distributed in a disordered form in amorphous soft domain. The rigid-rod crystals are assembled into a bundle-like structure in TPAE-0.39 as the increasing of polyamide content. The densely packed spherulites with feather-like structure can be seen in TPAE-0.10 with high polyamide content. These results are agreeable with the above deductions by AFM measurements.

### 3.4. Dynamic Thermomechanical Analysis

**Figure 9:**
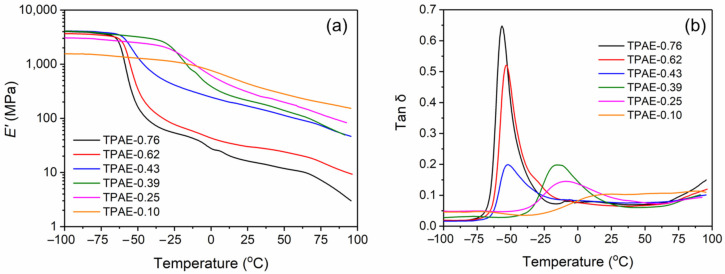
Thermomechanical analysis of TPAEs: (**a**) storage modulus (*E*’) vs. temperature and (**b**) loss factor (*tan δ*) vs. temperature.

To analyze the relationships between the microstructure and thermal mechanical properties of elastomers, DMA measurements were carried out on all the synthesized novel TPAEs. Figure 9 illustrates the temperature dependence of the storage modulus (*E′*) and loss factor (*tan δ*) for the TPAEs with variable compositions. The glass transition temperature *T_g_* (peak value of *tan δ*) and *E’* at −25 °C and 25 °C are listed in Table 2, correspondingly. It can be found that the copolymers have decreased *E’* with decreasing soft content at low temperature ca. −70 °C and exhibit a plateau of *E’* in the glassy state. Then, *E’* drops with the glass transition behavior. The *E’* values of TPAE-0.76 and TPAE-0.62 at −25 °C are far less than those of the other three samples, which are lower than commercial PEBAX at the similar compositions [5]. That means that these two copolymers with high soft content are well flexible at low temperature. Following the transition, the copolymers exhibit a rubbery state and a low dependence on temperature, providing elastic performance. The *E’* value at room temperature increases with the decreasing of soft content, which indicates the corresponding stiffness increases. It is worth noting that the *E’* value of TPAE-0.43 is smaller than that of TPAE-0.39, while the soft contents of two TPAE are very close. The *E’* of TPAE-0.43 has little change from −25 to 25 °C. To obtain TPAE with high elastic property, in addition to considering increasing the soft content, it is more significant to apply long-chain soft segments.

Figure 9b shows the thermal transitions of the TPAEs. The peak values of *tan δ* decrease with the increasing of polyamide content, indicating that polyamide crystals restrain the chain mobility of amorphous PPG. This is because the polyamide domain can act as a physical crosslinker in TPAEs [45]. The copolymers containing long-chain soft segments exhibit similar *T_g_* values that are independent of the soft content, whereas those having short-chain polyetheramine show increasing *T_g_* values as the soft content increases. The DMA results are in good agreement with the DSC results. The values of *T_g_* measured by DMA are higher than those by the DSC method, which is because the copolymer chain begins to relax at the temperature before the *tan δ* peak, which is measured by mechanical response [46].

*Tan δ* is defined using the ratio of loss modulus (*E’’*) and storage modulus (*E’*) and is used as an assessment of the ability of energy dissipation by elastomers. The strain hysteresis caused by the viscoelasticity of copolymers can result in energy absorption. Therefore, TPAEs are widely used as damping materials for attenuating vibration and reducing noise due to their significantly viscoelastic properties. Damping materials should meet the requirement of *tan δ* > 0.3, and the corresponding temperature region is defined as the damping temperature range [47,48]. The *tan δ* peak values of PEBAX are reported to be lower than 0.16, which is indicative of good energy return when using as an elastomer [5]. However, the *tan δ* peak values of the synthesized TPAEs are over 0.14 except for TPAE-0.10. Especially, the *tan δ* peak values of TPAE-0.76 and TPAE-0.62 with high PPG content are distinctly high (>0.5), which indicates that these two TPAEs have excellent shock absorbing capacity. It is because the energy dissipation by internal friction can be promoted since this C3 building block bears a methyl side group in the backbone [48]. As *tan δ* > 0.3, the damping temperature range for TPAE-0.76 and TPAE-0.62 are from −61 to −47 °C (ΔT = 14 °C) and from −58 to −45 °C (ΔT = 13 °C), respectively. It can be found that the synthesized TPAEs have excellent viscoelastic properties and damping performance by introducing a PPG block, which is important for toughness applications such as ski boots and rail gasket in an extremely cold situation. Thus, the novel TPAEs with long-chain semicrystalline polyamide and a high content of amorphous polyetheramine enrich the family of TPAEs for damping materials.

### 3.5. Mechanical Properties

**Figure 10:**
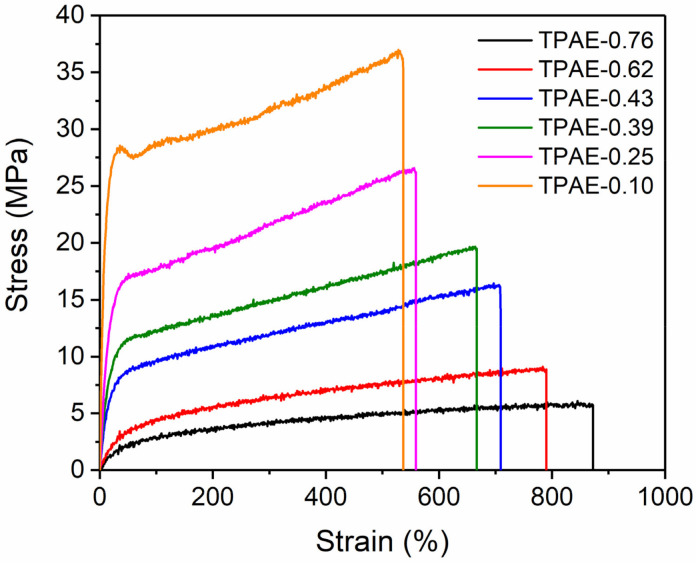
Tensile stress–strain curves of TPAE films.

Figure 10 presents the uniaxial tensile stress–strain curves of the TPAEs with different compositions at room temperature. The typical elastic behavior of the specimen is determined by the lack of a yield point during the deformation except for TPAE-0.10, which has the highest hard content and shows thermoplastic character. The polyetheramine-based TPAEs have good elastic properties according to the manner in which TPAE-0.25 reveals an intermediate stress–strain behavior. The Young’ moduli (the slope of initial linear elastic domain) increase with increasing polyamide content due to the increase in interconnectivity within the rigid polyamide domain [49]. Moreover, the tensile strength increases as the rigid content increases, which is independent of the polyetheramine length. The specimens exhibit good elongation at break that increases with the increase of soft content. Based on the testing results, the tensile property of TPAEs highly depends on the soft content instead of the segment length.

**Figure 11:**
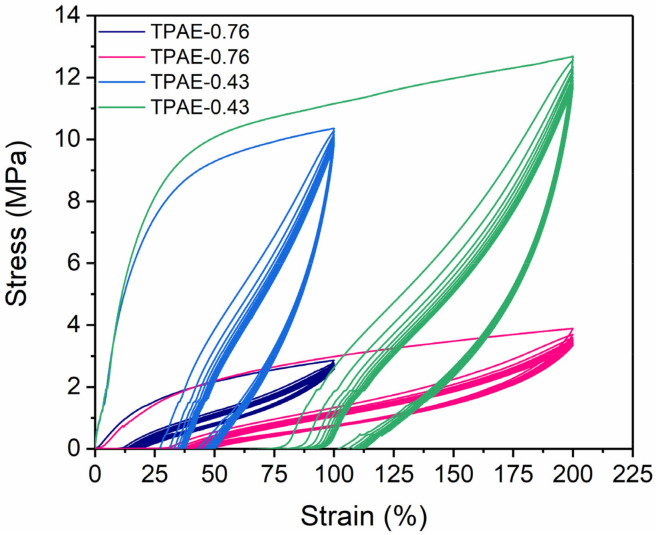
Selected cyclic tensile stress–strain curves of TPAE films.

To further study the elastic properties of the TPAEs, mechanical hysteresis was measured by a cyclic tensile test and the representative stress–strain curves are presented in Figure 11. The residual strain was calculated after 10 cycles of tensile testing. As we expected, the residual strain increases with the decreasing of soft content and increasing of testing deformation (Table S1, Supplementary Material). The novel TPAEs using diamine-terminated PPG present good elastic recovery and flexibility. In fact, the specimens after testing just have a small extent of residual strain after storing at room temperature overnight (Figure S4, Supplementary Material).

## 4. Conclusions

Novel TPAEs consisting of long-chain semicrystalline PA1212 (C12) as the hard segment and amorphous diamine terminated PPG (C3) as the soft segment were successfully synthesized via one-pot melt polycondensation. The chemical structures of the copolymers were characterized by NMR and FT-IR, and the results prove that the diamine terminated PPG segments have been incorporated to PA1212 blocks with expected composition. The molecular weights of the TPAEs were approaching the commercial grade. The microphase-separated morphologies and tensile properties of novel TPAEs have been systematically investigated by DSC, X-ray diffraction, AFM, SEM, and DMA. It has been found that microphase separation occurs in the amorphous region, and short-chain PPG diamine is miscible with the amorphous phase of PA1212. The copolymers adopt the same α-crystal phase as homo PA1212, and the crystallinity of the copolymers (PA1212 segments) varies with the content and segment length of PPG diamine. The microphase separation structure, associated with the macro properties, is tunable according to the contents and length of the flexible PPG diamine. Due to the amorphous PPG segment and long-chain semicrystalline PA1212, the new family of TPAEs shows high elasticity and damping performance, especially at low temperature for TPAE-0.76 and TPAE-0.62. The novel TPAEs show balanced properties of tremendous lightness, elastic return, and attenuation of vibration, suggesting their potential applications such as running shoes, ski boots, and tennis rackets even products used in extreme cold condition such as rail gaskets.

## Figures and Tables

**Table 1:** Compositions, GPC results, [η], density of TPAEs.

Sample Code	Feed Ratio of DA/DN/Jeffam (mol/mol)	MW of Jeffam (g/mol)	Calculated MW of PA1212 Segment (g/mol)	[η] (dL/g)	Density (g/cm^3^)	Soft Content in feed	Soft Content by NMR	M_n_ ^a^ (×10^4^) (g/mol)	M_w_ ^a^ (×10^4^) (g/mol)	PDI ^a^
TPAE-0.76	2/1/1	2000	624	1.26	1.01	0.77	0.76	2.34	5.48	2.34
TPAE-0.62	3/2/1	2000	1018	1.39	1.01	0.66	0.62	3.02	7.87	2.61
TPAE-0.43	8/7/1	2000	2988	1.36	1.02	0.45	0.43	3.82	8.83	2.30
TPAE-0.39	2/1/1	400	624	1.32	1.03	0.40	0.39	3.55	7.56	2.13
TPAE-0.25	3/2/1	400	1018	1.43	1.02	0.27	0.25	4.50	11.09	2.47
TPAE-0.10	8/7/1	400	2988	1.17	1.02	0.12	0.10	3.75	9.22	2.46

^a^ Determined by GPC.

**Table 2:** DSC and DMA results of TPAEs.

Sample Code	*T_m_* (°C)	*ΔH_m_* J/g	*T_c_* (°C)	Δ*H_c_* J/g	X_c_ (%)	*T_g_* (DSC) (°C)	*T_g_* (DMA) (°C)	*E’* at −25 °C (MPa)	*E’* at 25 °C (MPa)
TPAE-0.76	170.8	6.65	133.7	6.88	9.5	−60.3	−56.4	54.0	16.3
TPAE-0.62	177.4	16.06	137.9	16.22	14.5	−60.1	−53.6	77.4	30.0
TPAE-0.43	176.9	37.65	147.1	36.67	22.6	−60.8	−51.8	414.4	168.7
TPAE-0.39	156.2	35.00	111.2	35.37	19.6	−19.5	−15.7	2378.1	213.8
TPAE-0.25	163.3	43.48	127.9	43.46	19.8	−10.2	−9.2	2008.5	306.7
TPAE-0.10	176.8	54.03	140.6	55.70	20.5	N.D.	N.D.	1154.1	436.5

N.D. means not determined.

## Data Availability

The data presented in this study are available on request from the corresponding author.

## Supplementary Materials

The following are available online at https://www.mdpi.com/article/10.3390/polym13162645/s1, Figure S1: DSC thermogram of Jeffamine D2000, Figure S2: DSC thermogram of TPAEs, Figure S3: POM images of (a)(b) TPAE-0.39, (c) TPAE-0.25 (d) TPAE-0.10, (e) homo PA1212, Figure S4: Photos of specimens before and after cyclic tensile testing for (a) TPAE-0.76 and (b) TPAE-0.43 (stored at room temperature overnight), Figure S5: Thermal gravimetric curves of TPAEs, Table S1: The residual strain of the specimens after cyclic tensile testing, Table S2 Characteristic thermal degradation parameters of TPAEs.
