# Supplementary material for: Facile Synthesis of Thermoplastic Polyamide Elastomers Based on Amorphous Polyetheramine with Damping Performance
Source: Polymers (Basel). 2021 Aug 9;13(16):2645. doi: 10.3390/polym13162645 (PMC8397970; doi:10.3390/polym13162645)
Supplement: Supplementary file 1 [file polymers-13-02645-s001.zip › polymers-1311206-supplementary.pdf]

Supporting Information

Facile synthesis and characterization of thermoplastic polyamide elastomers based on semicrystalline PA1212 and amorphous polyetheramine

Jie Jiang[a], Qiuyu Tang[a], Xun Pan[b], Jinjin Li[a], Ling Zhao[a, c], Zhenhao Xi[a] and Weikang Yuan[a]*

[a] State Key Laboratory of Chemical Engineering, School of Chemical Engineering, East China University of Science and Technology, Shanghai 200237, China;

[b] Flinders Institute for Nanoscale Science and Technology, Flinders University, Sturt Road, Bedford Park, SA 5042, Australia

[c] College of Chemistry and Chemical Engineering, Xinjiang University, Urumqi 830046, Xinjiang, China

Corresponding Author: Prof. Zhenhao Xi
Email: zhhxi@ecust.edu.cn
Postal Address: Campus Box 369, No. 130 Meilong Road, Shanghai 200237

1. Measurement

Polarized optical microscopy (POM). The crystallization morphology of all samples was conducted on polarized optical microscopy (POM) apparatus (Olympus BX51, Japan) with a digital hot plate and a CCD camera. Samples were sandwiched between two glass slides with a gentle press. Samples were melted and pressed at 210 ^{o}C for 5 min to eliminate any thermal history, and then rapidly cooled to the designated temperature 145 ^{o}C at a rate of 50 ^{o}C min^{-1} and kept isothermal at the temperature for 15 min. The homo PA1212 was employed for comparison.

Thermal Gravimetric Analysis (TGA). Thermal stability of samples was studied on a NETZSCH TG 209 F1 (Germany) by heating a sample with around 5 mg initial weight from 25 ^{o}C to 600 ^{o}C at a rate of 10 ^{o}C min^{-1} under nitrogen atmosphere.

2. Results and discussion

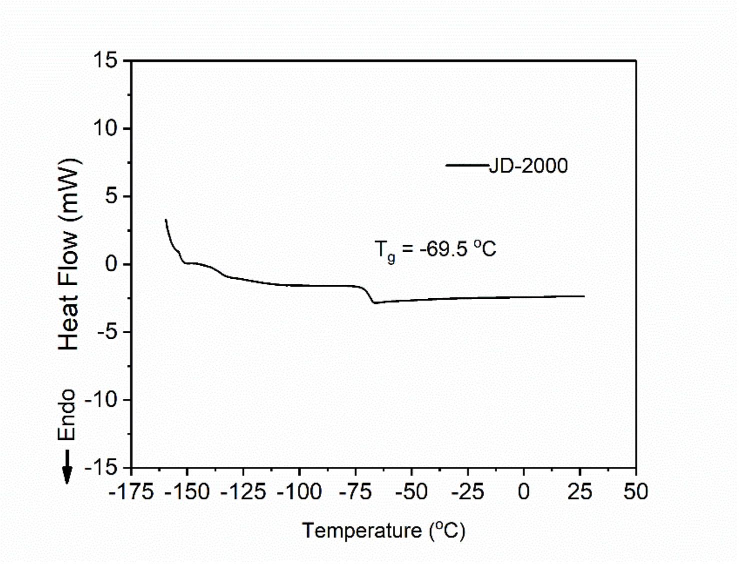

Figure S1. DSC thermogram of Jeffamine D2000.

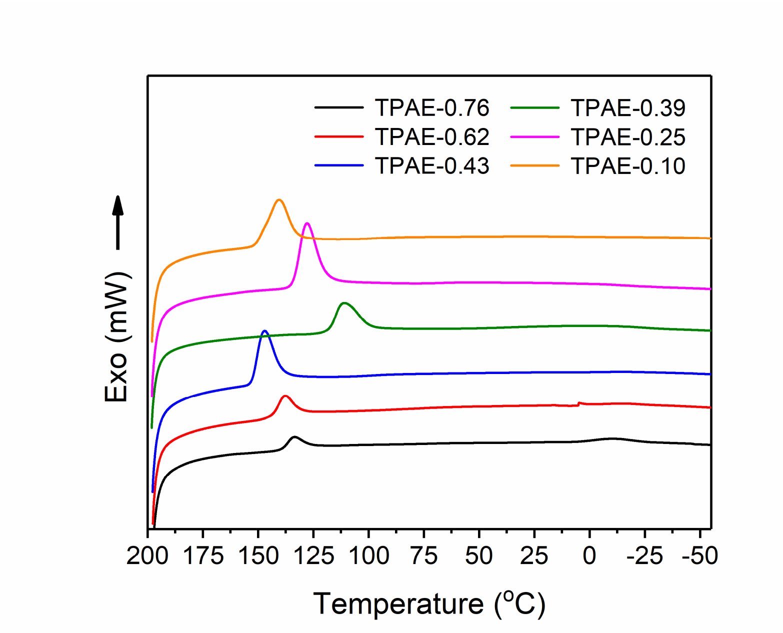

Figure S2. DSC thermogram of TPAEs.

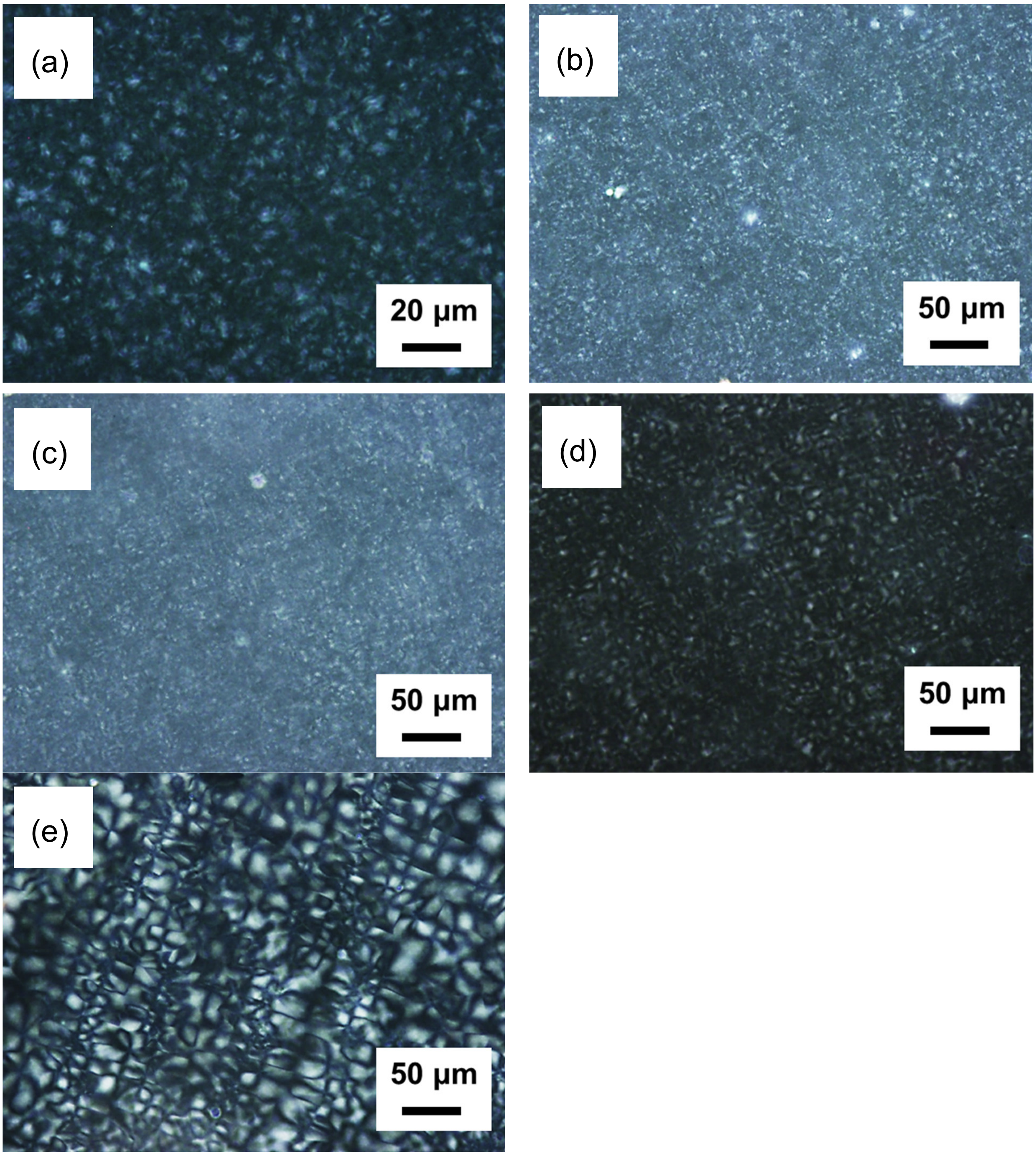

Figure S3. POM images of (**a**,**b**) TPAE-0.39, (**c**) TPAE-0.25 (**d**) TPAE-0.10,. (**e**) homo PA1212

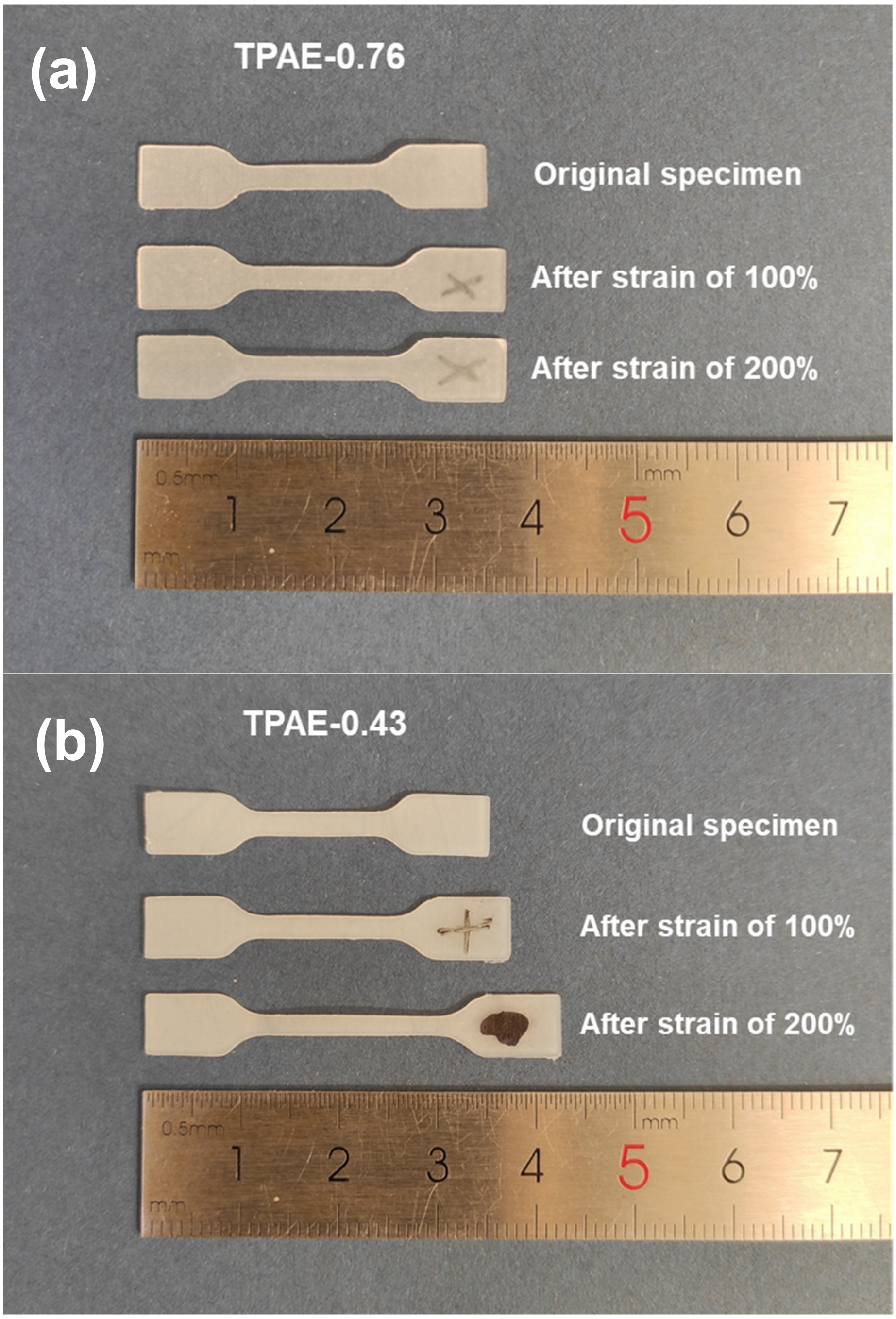

Figure S4. Photos of specimens before and after cyclic tensile testing for (**a**) TPAE-0.76 (**b**) TPAE-0.43 (stored at room temperature overnight).

Table S1. The residual strain of the specimens after cyclic tensile testing.

	Strain	
	100%	200%
TPAE-0.76	16%	41%
TPAE-0.43	30%	94%

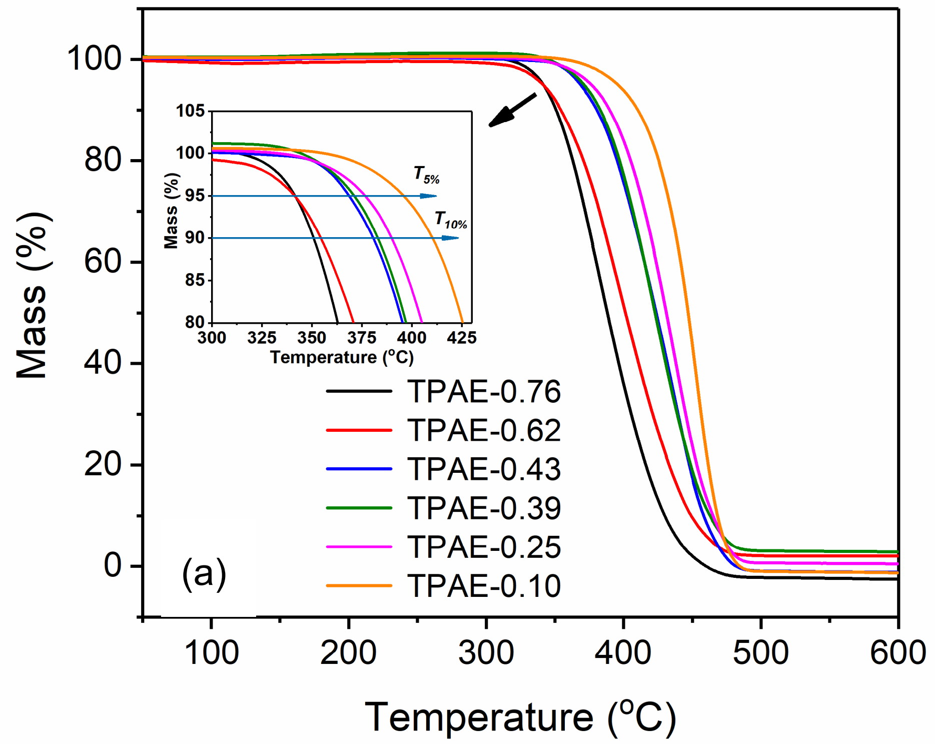

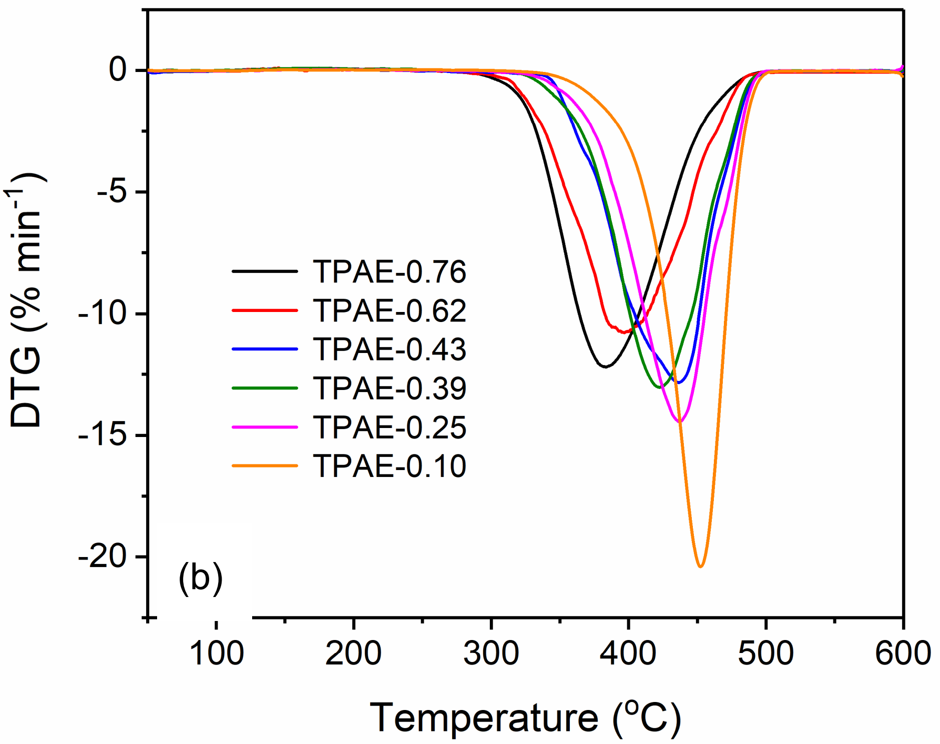

Figure S5. Thermal gravimetric curves of TPAEs.

TGA was employed to study the thermal stability of TPAEs and the corresponding curves are shown in Figure S5. The

temperatures at 5% of decomposition ($T_{5\%}$), 10% of decomposition ($T_{10\%}$), and maximum decomposition temperature (T_{max}) of the TPAEs are listed in Table 3. The initial decomposition temperature ($T_{5\%}$) of all samples are above 340 ^{o}C that is far over the processing temperature ca. 210 ^{o}C. Generally, as the polyamide content increases, the $T_{5\%}$, $T_{10\%}$ and T_{max} increase. The thermal stability of the TPAEs containing PPG segment (C3) is lower than that using PPDO (C3) as soft segment because the tertiary carbons are prone to break up under high temperature. The six copolymers display one-step decomposition procedure. It appears that the copolymers with high amount of soft segment have relatively low maximum decomposition temperature although the thermal stability of pristine polyetheramine is poorer than that of homo PA1212. These results reveal that diamine terminated PPG based TPAEs can keep good thermal stability in processing.

Table S2. Characteristic thermal degradation parameters of TPAEs.

Sample code	$T_{5\%}$ (^{o}C)	$T_{10\%}$ (^{o}C)	T_{max} (^{o}C)
TPAE-0.76	341.2	350.7	384.2
TPAE-0.62	341.7	354.4	397.4
TPAE-0.43	368.5	380.9	436.3
TPAE-0.39	371.3	382.9	422.6
TPAE-0.25	376.4	389.9	437.1
TPAE-0.10	395.9	410.4	452.4
